# Genetics of spontaneous cervical and coronary artery dissections

**DOI:** 10.3389/fgwh.2023.1007795

**Published:** 2023-05-05

**Authors:** Isabel Rada, Juan Francisco Calderón, Gonzalo Martínez, Paula Muñoz Venturelli

**Affiliations:** ^1^ Instituto de Ciencias e Innovación en Medicina, Facultad de Medicina Clínica Alemana Universidad del Desarrollo, Santiago, Chile; ^2^ División de Enfermedades Cardiovasculares, Pontificia Universidad Católica de Chile, Santiago, Chile; ^3^ The George Institute for Global Health, Faculty of Medicine, University of New South Wales, Sydney, NSW, Australia

**Keywords:** genetics, genetic variation, cervical artery dissection, coronary artery dissection, vascular disease in women, genetic association, genetics of artery dissections

## Abstract

**Objectives:**

Spontaneous cervical artery dissections (SCeAD) and coronary artery dissections (SCoAD) are major causes of neurovascular and cardiovascular morbidity in young adults. Although multiple aspects of their etiology are still unknown, most consensuses are focused on the presence of constitutional genetic aspects and environmental triggers. Since recent evidence of genetic contribution points to a possible overlap between these conditions, we aimed to describe current information on SCeAD and SCoAD genetics and their potential shared pathological aspects.

**Materials and methods:**

A narrative review is presented. Publications in English and Spanish were queried using database search. The articles were evaluated by one team member in terms of inclusion criteria. After collecting, the articles were categorized based on scientific content.

**Results:**

Given that patients with SCeAD and SCoAD rarely present connective tissue disorders, other genetic loci are probably responsible for the increased susceptibility in some individuals. The common variant rs9349379 at *PHACTR1* gene is associated with predisposition to pathologies of the arterial wall, likely mediated by variations in Endothelin-1 (ET-1) levels. The risk of arterial dissection may be increased for those who carry the rs9349379(A) allele, associated with lower expression levels of ET-1; however, the local effect of this vasomotor imbalance remains unclear. Sex differences seen in SCeAD and SCoAD support a role for sex hormones that could modulate risk, tilting the delicate balance and forcing vasodilator actions to prevail over vasoconstriction due to a reduction in ET-1 expression.

**Conclusions:**

New evidence points to a common gene variation that could explain dissection in both the cervical and coronary vasculatures. To further confirm the risk conferred by the rs9349379 variant, genome wide association studies are warranted, hopefully in larger and ethnically diverse populations.

## Introduction

Arterial dissections are characterized by the separation of the arterial wall layers, creating an intramural hematoma either between vessel intima and media or media and adventitia. This can impair blood flow and adequate tissue perfusion, leading to ischemia and organ failure, and eventually cause death (1). Recently, there has been a renewed interest in spontaneous cervical artery dissections (SCeAD) and coronary artery dissections (SCoAD), due to improved diagnosis and the recognition that ischemic events derived from them can have severe clinical consequences (2, 3). Both types of artery dissections affect young populations and are particularly important in women since they occur more frequently during pregnancy and postpartum (4).

The pathological mechanisms behind SCeAD and SCoAD have still to be clarified. Although their presentation is mostly sporadic (5, 6), consensus exists regarding the presence in these patients of a constitutional genetically determined weakness of the vessel wall that, combined with triggering environmental factors, such as acute infection, minor trauma or physical stress, results in vessel injury (7–9). An underlying vasculopathy is suggested by the fact that patients with SCeAD and SCoAD commonly present with concomitant arterial anomalies, such as fibromuscular dysplasia (FMD) (1, 10). Classically considered distinct entities, recent evidence describes possible genetic associations for both types of dissections (11, 12). Our aim is to describe the current literature on SCeAD and SCoAD genetics and the potential shared mechanisms underpinning these two conditions.

## Methods

The studies included in this narrative review (Supplementary Table eS1) were identified by searching in the electronic databases PubMed and ScienceDirect. Keywords were related to the pathological conditions and genomic/genetic terms “cervical artery dissection”; “carotid artery dissection”; “coronary artery dissection”; “mRNA”; “single nucleotide variant”; “single nucleotide polymorphism”; “polymorphism”; “genetic”; “genomic” using Boolean connectors AND/OR respectively. The search was restricted to manuscripts published from 1950 to present published in English. The selection criteria were as follows: (i) type of study: case studies, case-control studies, cohort studies; (ii) population type: diagnosis of SCeAD or SCoAD, without age or comorbidities restrictions; (iii) type of measurements: biological confirmation through genetic testing; (iv) type of outcomes: genetic/genomics reports, e.g.,: copy number variants (CNV), single nucleotide variants (SNV) and microRNA.

## Results

### Epidemiology and clinical implications of spontaneous cervical and coronary dissections

While SCeAD has an annual incidence rate of 2.6–2.9 cases per 100,000 people (13), official epidemiological data from SCoAD is not so well documented (14). There is a sex predominance for SCoAD, affecting more women (81%–92%) (15). Conversely, it appears that SCeAD has a slight tendency for higher cases in males (53%–57%), with the caveat that this estimated predominance can be biased because SCeAD patients with local symptoms might go under-recognized (13, 16). The overall population affected by these dissections is classically young-middle aged adults with scarce cardiovascular risk factors. The mean age of onset for SCeAD is 45 years (13); similarly, SCoAD reports a mean age ranging between 42 and 53 years (15, 17).

Both dissections can lead to severe clinical consequences. SCeAD accounts for 10%–25% of all stroke events in young patients (13) with increased risk within the first 2 weeks after dissection (18). Likewise, SCoAD can lead to myocardial injury and infarct, causing 1%–4% of all acute coronary syndromes and it has been associated to 0.5% of sudden cardiac deaths, particularly in young individuals (7, 19).

The occurrence of each condition is related to different environmental triggers. While SCeAD associates with minor cervical trauma (20), seasonal peak during winter (21), recent acute infection and migraine (11), SCoAD has been related to physiological and physical stress (e.g., strenuous exercise), leaving aside traumatic, iatrogenic or atherosclerotic etiologies (7–9). Nonetheless, SCeAD and SCoAD share common risk factors, including connective tissue disorders (CTD), FMD, and pregnancy/postpartum (8, 20, 22, 23), as it will be discussed later.

Regarding sex differences, the clear predominance of SCoAD in women is particularly important since this entity represents the major cause of myocardial infarction in pregnant women, particularly during the third trimester or early postpartum (14). Likewise, a series of SCeAD cases have been reported involving postpartum women, not only related to the stress of vaginal delivery but also after cesarean section (4). Estrogen, a steroid hormone whose most active metabolite is 17β-estradiol, increases its levels among adolescence, pre-menopause women and fluctuates and drops at older ages (24). Estrogens have vasodilatory effects throughout the activation of its receptors, promoting the release of nitric oxide in endothelial cells by activation of the PI3-kinase/AKT pathway (25); as well as endothelial prostacyclin (PGI2) production via arachidonic acid derivatives (26, 27). Additionally, the action of estrogens on the inhibition of the vasoconstrictor role of the renin-angiotensin-aldosterone system has been shown, favoring vasodilatation by increasing natriuretic peptide (28, 29). Also, estradiol has an inhibitory effect on endothelin 1 (ET-1) synthesis, further favoring a vasodilatory phenotype (30–32). Also, preclinical studies have suggested that pregnancy can increase the expression of oxytocin receptors in vascular tissue and can influence the risk of dissection. Previous research in murine models with CTDs showed that oxytocin levels are involved in extracellular signal-regulated kinase (ERK) pathway activation inducing aneurysm progression. This was confirmed by dissection reduction after suppression of lactation stimulus, ERK inhibition and oxytocin antagonist treatment (33, 34).

Thanks to the improved recognition and more precise diagnosis of these conditions by clinicians during the last couple of decades, a gradually more robust data have been accumulating that hopefully will help to better describe the role of modulating factors and environmental triggers, which end up resulting in arterial dissection.

### Fibromuscular dysplasia, spontaneous cervical and coronary dissection

FMD is defined as “an idiopathic, segmental, non-atheromatous disease of the musculature of arterial walls, leading to stenosis of small and medium-sized arteries” (35). The presence of FMD has been described in series of both SCeAD and SCoAD patients, ranging between 5%–31% and 45%–86%, respectively (1, 36–40). Several epidemiological reports of US and European origin point to a preponderance of females in FMD cases, mostly diagnosed at age 50–55 years (41, 42), highlighting epidemiological similarities with SCoAD, where FMD is believed to be the underlying arteriopathy in the majority of cases (43). Considering the possible association between FMD, SCeAD and SCoAD, it has been suggested that these disorders comprise clinical manifestations of a common pathological entity (44). Beyond its association to vessel dissection, FMD in the renal arteries can lead to arterial hypertension, further increasing cardiovascular risk.

Recently, systematic recognition of FMD has been performed in large prospective cohort studies. For example, among 750 SCoAD patients, the overall presence of FMD was identified in 31.1% (39). Specifically, 56.7% (233/411) of patients with complete screening had FMD (39). Meanwhile, the screening of 1,283 SCeAD resulted in 8.0% of FMD diagnosis (103/1,283). Of note, the cerebrovascular FMD was detected as a potential marker of recurrence (40). However, a delay in FMD diagnosis and incomplete screening in SCeAD and SCoAD patients might favor the underestimation of the shared prevalence between these conditions.

**Figure 1:**
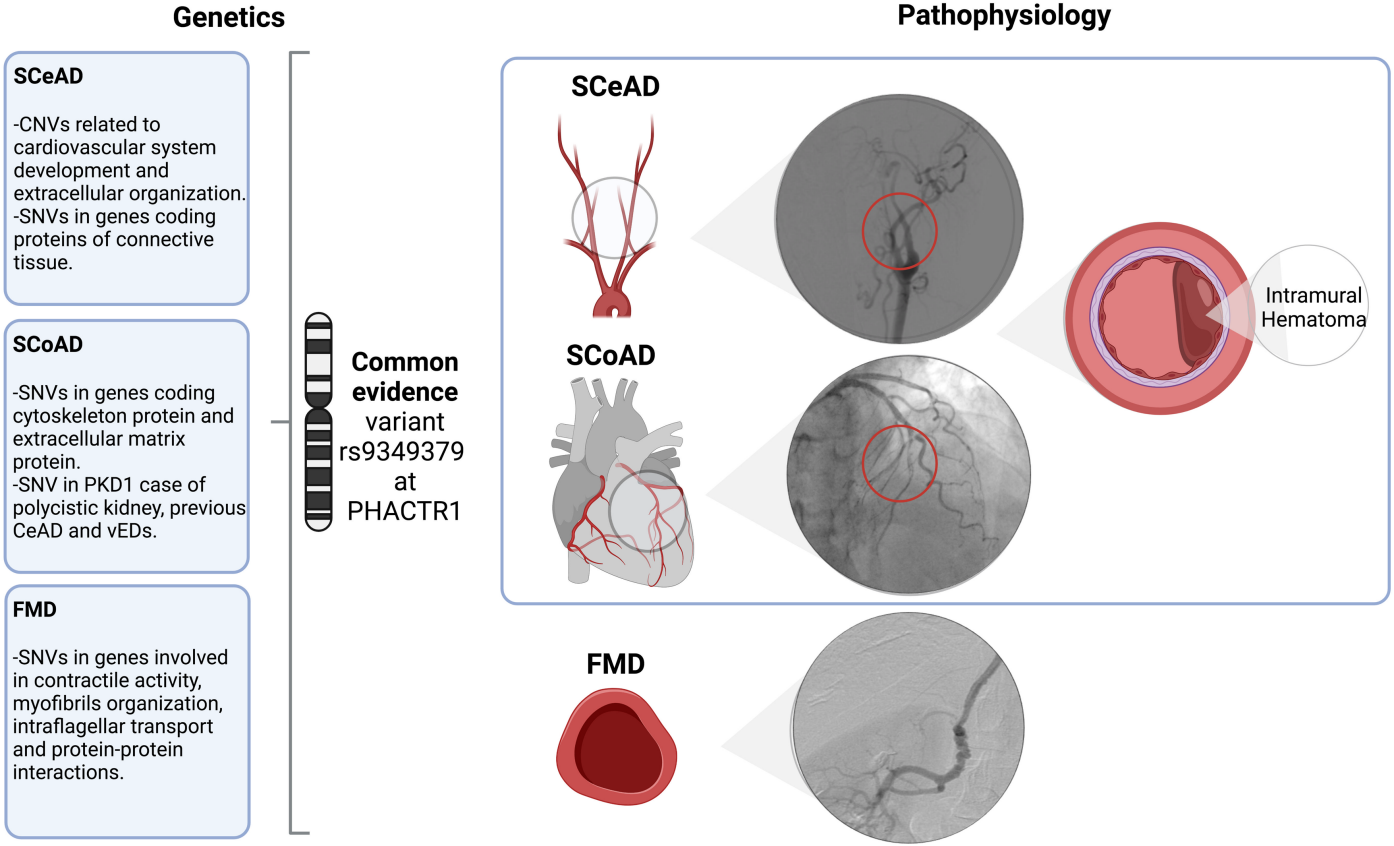
Overview and associated arteriopathy of spontaneous cervical and coronary dissection and fibromuscular dysplasia. SCeAD, SCoAD and FMD have been previously associated as clinical manifestations of a common pathological entity. Though, literature reported specific genetic contribution and its functional correlation for each condition, the common variant rs9349379 at *PHACTR1* might represent a potential shared underlying mechanism. Specifically, SCeAD and SCoAD results from the separation of arterial wall layers producing an intramural hematoma with or without intimal tear. A low frequency of SCeAD, SCoAD and FMD cases are related to connective tissue disorders. SCeAD, spontaneous cervical artery dissection; SCoAD, spontaneous coronary artery dissection; FMD, fibromuscular dysplasia; CNVs, copy number variants; SNVs, single nucleotide variants; PDK1, polycystin-1; vEDs, vascular Ehlers-Danlos syndrome.

FMD appears to be much more prevalent than previously thought. For example, silent FMD has been recognized in up to 6% of potential kidney donors (45, 46). While renal and cervico-cephalic arteries are the most commonly affected vascular beds, almost half of patients can have multivessel compromise, including visceral, ilio-femoral and coronary arteries (47). Furthermore, a familial presentation of FMD has been previously described (48–50), however, this association is now believed to be uncommon. In the ARCADIA study, familial FMD was present in 2.9% of cases (47); thus, most cases are sporadic. Nevertheless, as genetic background is highly probable, potential gene variants have been proposed. Using exome sequencing, the presence of multifocal FMD was associated to myosin light chain kinase (*MYLK* – also involved in thoracic aneurysms), dynein cytoplasmic heavy chain 1 (*DYNC2H1*), sarcomeric protein obscuring (*OBSCN*), and *RNF213* (involved in Moyamoya disease) genes (42). Additionally, (51) Kiando et al. 2017 found that allele A of a genetic variant (rs9349379) of the phosphate and actin regulator 1 gene (*PHACTR1*) was associated to a 40% increase in the relative risk of FMD (51). As it will be discussed later, this gene is also associated with vascular dissections (Figure 1).

### Spontaneous cervical artery dissection genetic studies

The CTDs monogenic disorders have been explored in regard to the predisposition to SCeAD. Autosomal dominant conditions such as Marfan syndrome (MFS) caused by variants in *FBN1* gene (52), vascular Ehlers-Danlos syndrome (vEDS) caused by variants in *COL3A1* (53) and Loeys-Dietz syndrome (LDS) caused by variants in *TGFBR1*, *TGFBR2* and *SMAD3* (54, 55), among other genes, have been considered as potential disorders favoring SCeAD (3, 56). However, a comprehensive systematic review in 2009 reported low frequency of these heritable cases, showing that vEDS accounted for only 2% of all SCeAD cases, while in MFS the overall reported cases were less than 1% (10). Although these studies may have resulted in an underestimation due to the lack of a systematic diagnostic protocol and disease recognition mainly derived from clinical features without molecular confirmation, clinical registry data seem to support these findings, as an association with CTDs is low (56, 57). The systematic review also reported predominantly negative findings from 15 genetic association studies, although the candidate genes coding for intercellular adhesion molecule 1 (*ICAM-1*), collagen type III a-1 (*COL3A1*) and methylenetetrahydrofolate reductase (*MTHFR*) involved in regulation of extracellular matrix (ECM) were associated in five studies (58–62). Moreover, a meta-analysis based on evidence of *MTHFR 677TT* genotype yielded significant association with SCeAD; however, a subsequent case-control study elicited opposite evidence, where the genotypic frequencies for this variant and others linked to SCeAD, thrombosis, coronary artery disease, hypertension and lipoprotein levels did not differ with their control counterparts (63). Furthermore, analyses of other gene variants involved in ECM homeostasis, (promoter region of matrix metalloproteinases or MMPs), lacked statistical differences between individuals with SCeAD and controls (64).

Regarding the identification of causative copy number variants (CNVs) in SCeAD, a case-control study from patients with or without dermal tissue alterations and its comparison with control samples and earlier published data, retrieved 34 CNVs associated with SCeAD (65). Those affected with dermal tissue alterations showed higher density of CNVs and ontology analyses revealed enrichment for genes related to ECM and collagen fibril organization. Although authors found novel rare CNVs in SCeAD population (duplication *MYH11*/*ABCC6* and deletion *SGCZ*) that have also been described for aortic dissection, none of these were located in any other dissected participant (65). In line with exploration of CNVs, Grond-Grinsbach et al*.* used a Single Nucleotide Polymorphism (SNPs) array and genotyped a large set of SNPs in 883 patients. CNVs of SCeAD carriers covered 433 protein-coding genes associated to muscle organ development and cell differentiation of cardiovascular system; interestingly, the altered *MYH11* encoding for smooth muscle myosin heavy chain and *ABCC6* involved in membrane transportation were also found in SCeAD cases (3 patients) (66). Continuing with SNPs examination, the association between variants and arterial connective disorders was tested in SCeAD cases from 9 families with 2 affected members. The SNPs genotyping yielded 1,242 variants in the sample from which 142 were nonsense or missense substitutions. Among them, 9 were non-benign variants identified in 4 out of the 9 families. Specifically, these variants were found in *COL3A1*, *FBN1*, *COL4A1* and *TGFBR2* genes each in one of these 4 families. Although an increased susceptibility is suggested due to familial SCeAD, genetic heterogeneity makes difficult to establish a single causal variant, thus favoring a polygenic model (67).

**Figure 2:**
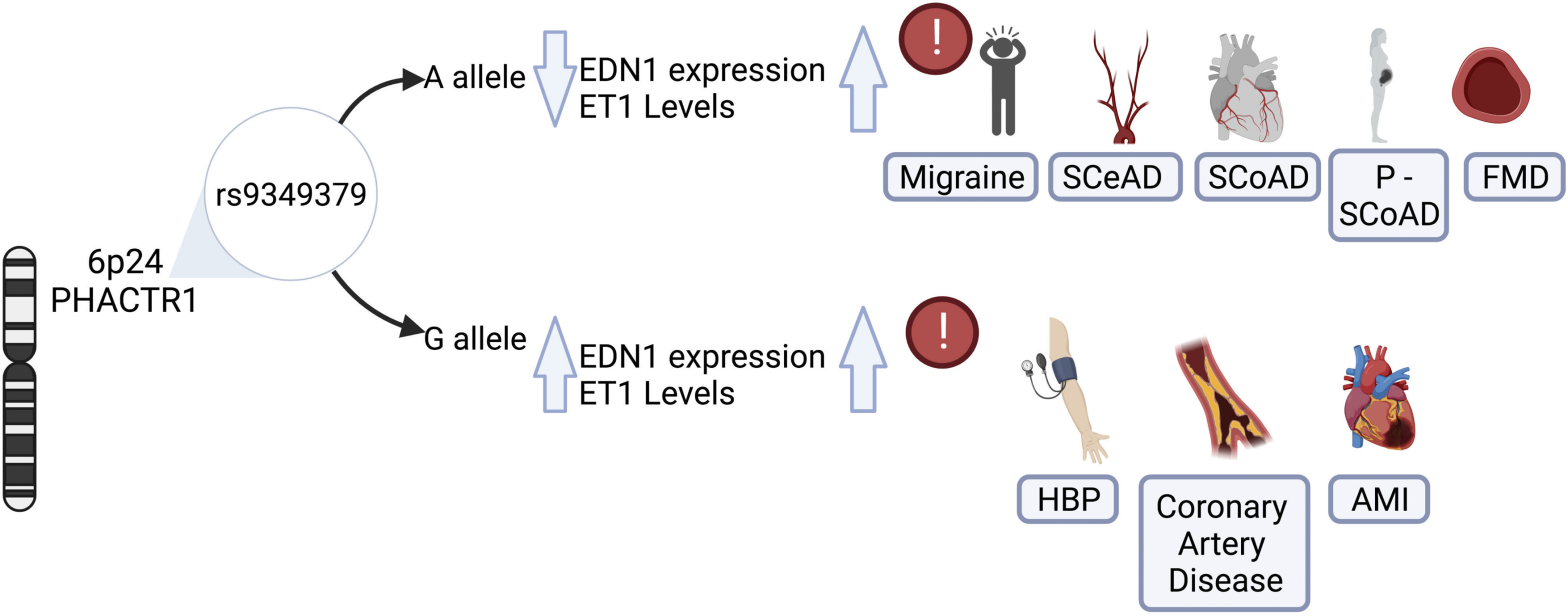
Role of rs9349379 genotypes on endothelin-1 levels and its risk associated with vascular diseases. The genetic variant rs9349379 in PHACTR1 gene regulars EDN1 gene expression, influencing protein levels and inducing its imbalance. The rs9349379 major allele (**A**) down regulate EDN1 gene expression, lowering ET1 secretion and favoring vasodilatation. Those carrying A allele are at risk for SCeAD, SCoAD migraine and FMD predominantly in the female population. Contrary to the effect G allele which increase ET1 levels that favors vasoconstriction and is associated with HBP, CAS, and AMI, mainly affecting the male population. EDN1, endothelin-1 gene; ET1, endothelin-1 protein; SCeAD, cervical artery dissections; SCoAD, spontaneous coronary artery dissections; P-SCoAD, pregnancy-associated SCoAD; FMD, fibromuscular dysplasia; HBP, high blood pressure; CAS, coronary artery disease; AMI, acute myocardial infarction.

More recently, an inverse association of a variant located in *PHACTR1* with SCeAD was recognized in a large collection of cases from European origin recruited in European and US centers (11). Specifically, a genome wide association analysis revealed an association of rs9349379 variant in *PHACTR1* with SCeAD during discovery and follow-up phases with the rs9349379(G) allele conferring lower risk of dissection (11) (Figure 2). This variant has been previously associated with myocardial infarction and coronary calcifications in various ancestry groups (68–71), with effects in the opposite direction of that in SCeAD. Conversely, a comparison between African American (AA) and European American (EA) population with stroke and SCeAD from the 1,000 genome project data, showed a higher allele frequency for rs9349379(A) in the AA group with SCeAD, but, paradoxically, AA had less SCeAD prevalence compared to EA (72). Of note, those from AA group with allele A also presented a greater rate of cardiovascular risk factors. These conflicting results could be explained by the existence of intermediate modulators of the effect of this variant, but also to bias of a single center database, where SCeAD can be underdiagnosed in low cardiovascular risk populations (72).

### Spontaneous coronary artery dissection genetic studies

Similarly to SCeAD, CTDs have historically represented the main focus of SCoAD genetic research, yet it has been identified as the causative disorder in less than 5% of these cases (73). Few case studies have reported the presence of vEDS-related variants in *COL3A1* gene, mainly in patients with multivessel events and/or SCoAD recurrence (74–77). Interestingly, two of these cases were pregnancy-associated SCoAD (74, 76). Studies referring patients to genetic analysis from larger SCoAD case series have led to the detection of a small proportion of participants with disease-causing variants. Henkin et al. (78), used diverse panels of genes associated with CTDs to test SCoAD-affected individuals, in which only 3 out of 59 - the total sample who underwent genetic testing - had variants either in *FBN1* or *COL3A1* (78). Similarly, a prospective cohort confirmed the presence of *COL3A1* variants in 3 patients and *SMAD3* in one case from subjects who agreed to the genetic evaluation. Noteworthy, one patient with variants in polycystin-1 (*PKD1*) - who was diagnosed with polycystic kidney disease - had a previous SCeAD, followed by a SCoAD four years after the first dissection event (79). The autosomal dominant polycystic kidney disease (ADPKD) might produce arterial wall weakening and increase susceptibility to vascular events. Evidence has documented that half of deaths among patients with ADPKD were produced by myocardial ischemia (80). Moreover, studies looking beyond traditional CTDs have reported the detection of rare variants in the gene encoding cytoskeletal protein Talin 1 (*TLN1*) in a familial SCoAD case composed of 5 members from which 3 had dissections. This variant was subsequently found in 10 cases of a large SCoAD cohort, thus, it is believed to have detrimental vascular effects, since TLN1 alteration has been also found in aortic dissection (81).

The association of SCoAD with the variant rs9349379 in *PHACTR*1 has been recently recognized. This variant was previously described for coronary diseases such as atherosclerosis and acute myocardial infarction (82–84). Adlam et al*.* (12) analyzed SCoAD cases and controls studies, along with pregnancy-associated SCoAD cases from the United States, United Kingdom, Australia and France with confirmed European Ancestry. In accordance with epidemiological data of SCoAD, the study revealed a female predominance (87%–96%), with pregnancy-associated SCoAD accounting for 10% of cases. Besides sex influence, based on more than 1,000 cases, it was estimated that those who carried the rs9349379-A allele had 70% greater risk of SCoAD. This was associated with lower plasma levels of Endothelin-1 (ET-1) according to measurements from a subset of SCoAD patients from the United Kingdom with similar characteristics (Figure 2) (12). Similarly, a meta-analysis of genome wide SCoAD discovery and replication reported an association with the variant rs9349379 in *PHACTR1* (85). In addition, the genome wide approach in a prospective large cohort of SCoAD patients revealed an association of the variant rs12740679 in *ADAMTSL4* gene chromosome 1q21.2, which encode an extracellular matrix protein involved in microfibril formation (85). Although the pathophysiology of SCoAD is not yet well understood, these latest findings give new insights for further research focused on elucidating the role of the common rs9349379 variant.

### Potential common underlying mechanisms in spontaneous cervical and coronary dissection

As previously mentioned, the common variant rs9349379 at *PHACTR1* locus on chromosome 6p24 has been proposed as a potential causative variant for pathologies of vascular phenotype, mostly described for chronic coronary diseases (84, 86, 87). However, the emerging evidence of other vascular disorders such as FMD (51), SCeAD (11, 72, 88) and SCoAD (12, 85) have raised the attention to explore the potential underlying mechanisms of this genetic locus. Gupta et al*.*, reported a common intergenic area between rs9349379 at *PHACTR1* locus and the promoter of endothelin-1 gene (*EDN1*), with putative regulatory function on the expression of these genes (89). Specifically, *PHACTR1* encodes for a protein of phosphatase and actin regulator family related to regulation of the actin cytoskeleton and, indirectly, on the endothelial cell survival (90). In turn, this could have deleterious effects in several vascular phenotypes such as formation of atherosclerotic plaques (91), neo-angiogenesis (92), cell proliferation and migration in cerebral microvasculature (93). Several experiments in stem cell lines reprogramed with CRISPR/Cas9 validated the influence of the genotype at rs9349379 on *EDN1* levels, in which the minor allele in homozygosity (G/G) resulted in higher *EDN1* expression, contrary to the reduced levels observed in association with homozygous major allele (A/A) (89). The association was confirmed in plasma samples from healthy individuals: those who carry the minor allele expressed higher levels of the precursor Big- ET-1 that is subsequently converted to the active form ET-1. The authors proposed a model where rs9349379 (G) confers risk for coronary artery disease/myocardial infarct, whereas it reduces susceptibility to SCeAD, FMD and migraine (58, 89). Given these findings, the major allele rs9349379 (A) may impose the risk genotype for both dissections, along with reduced ET-1 levels as described in a large sample of SCoAD patients (12). ET-1 is a peptide with potent vasoconstrictor action synthesized by different cell types, predominantly vascular endothelium. ET-1 activates receptors ET_A_ y ET_B2_ generating a vasoconstrictor effect mediated by calcium, while ET_B1_ promotes the release of vasodilator agents as nitric oxide and prostacyclin (94). Although the response of increased levels of ET-1 have been widely characterized as endothelial damaging (95), the vascular susceptibility that could result from reduced expression of *EDN1* has not been explored.

The findings of the present study may have relevant clinical implications for the prediction of dissection, based on genetic contribution described in the evidence. Particularly, populations carrying the major allele rs9349379 (A) at *PHACTR1* might have down regulated expression of *EDN1* and lower ET-1 levels. This imbalance favoring vasodilation and affecting vascular integrity, could also interplay with sex hormones. This can be critical for specific interest groups such as women at all stages of their life, particularly during pregnancy. Therefore, the study of the hormonal influence on arterial wall vulnerability could improve the understanding of female predominance in SCoAD and pregnancy-associated dissections.

## Conclusion

Emerging evidence has related vascular disorders such as SCeAD, SCoAD and FMD to the common variant rs9349379 at *PHACTR1*, likely mediated by ET-1 levels. The risk of arterial dissections may increase for those carrying the rs9349379(A) variant and thus express lower ET-1 levels; however, local effect or vascular remodeling resulting from this vasomotor imbalance remains unclear. Sex differences support a role for sex hormones on risk modulation, by which vasodilator actions might likewise prevail over-active vasoconstriction. Further research is needed to confirm the risk conferred by the rs9349379 variant, including genome wide association studies in larger and ethnically diverse SCeAD, SCoAD and FMD patients.
