# Supplementary material for: Genetics of spontaneous cervical and coronary artery dissections
Source: Front Glob Womens Health. 2023 May 5;4:1007795. doi: 10.3389/fgwh.2023.1007795 (PMC10196206; doi:10.3389/fgwh.2023.1007795)
Supplement: Supplementary file 1 [file Table1.docx]

**Supplementary material**

Table e1. Summary of main evidence on genetics of spontaneous cervical artery dissections and coronary artery dissections.

| Author | Population | Main findings |
| --- | --- | --- |
| Saw et al. 2020 (76) | Discovery study  SCAD cases from CanSCAD study n=270  M=29  F=241  Age: 53.3 ± 9.7 years    Matched CTL from Michigan Genomics Initiative biorepository n=5.263  Replication study  CanSCAD n=163    Matched CTL n= 3.207 | rs12740679 in *ADAMTSL4* was associated with SCAD at discovery phase and replication. The meta-analysis of discovery and replication results revealed associations between SCAD with rs11172113 in *LRP1* and rs9349379 in *PHACTR1*. |
| Turley et al. 2019 (71) | Familial SCAD members n=5  (5 living relatives)  Sporadic SCAD cases n=56 (out of 675)  CTL n=291 | *TLN1* was the top candidate gene. The WES analyses in familial and sporadic SCAD cases revealed a rare heterozygous missense variant in *TLN1*.  *TLN1* rare variant with high probability of loss of function intolerance. *TLN1* variant is overrepresented in SCAD vs. CTL. |
| Adlam et al. 2019 (75) | European Ancestry SCAD patients n= 1.005  CTL n=7.190    French cases n=189  M=19 F= 170  Age: 51 (44–59)  French CTL n=3.964  M=2.952   F=1.012  Age: 58.73 ± 5.94 years    United Kingdom cases n=202  M=8 F=194  Age: 46 years (42–53)  United Kingdom CTL n=606  M=24 F=582  Age: 44 years (44–44)    Australian cases n=160  M=6 F=96  Age: 50 years (45–57)  Australian CTL n=1.127  M=455 F=672  Age: >75 years    Mayo Clinic cases n=504  M=22  F=482  Age: 48 years (41–55)  Mayo Clinic CTL n=1.493  M=70 F=1.423  Age: 48 years (41–55)    Meta-analysis cases  Acute myocardial infarction (AMI) n =43.171  Female with coronary artery disease (CAD) n=9.105  Male with coronary artery disease n=30.428  CTL n=127.176  Female n=30.428  Male n=36.042 | CAD cases showed higher prevalence and frequency of the allele rs9349379(A) in *PHACTR1* vs. CTL. In addition, there was a significant association between this allele and the risk for SCAD.  Among pregnancy and recurrent cases there were no differences in allele distribution.  The combined meta-analysis revealed an odds ratio of 1.67 (95% CI: 1.50-1.86) per risk allele increment. The effect was opposite for AMI/CAD cases (OR: 0.88; 95% CI: 0.86-0.89) and analysis restricted to women (OR: 0.92; 95%CI: 0.88-0.96) |
| Kaadan et al. 2018  (65) | SCAD patients n = 44 with genetic testing (out of 73)  M=11 F=35  Age: 43.6 ± 9.6 years | There were pathological mutations in *COL3A1* (n=3 patients), *PKD1* (n=1 patient), *SMAD3* (n=1 patient), *LMX1B* (1 patient). In addition, variants of unknown significance were found in 12 patients. |
| Green et al. 2018 (60) | CeAD and ischemic stroke patients (major trauma etiology was excluded) n = 266  African Ancestry CeAD patients (AA)  n =20  M=11 F= 9  Age: median 43 years    European Ancestry CeAD patients (EA)  n= 173  M= 76 F=97  Age: median 44 years | AA group had less prevalence of CeAD compared to EA.  AA group showed significantly higher allele frequencies for rs9349379(A) in the PHACTR1 vs. EA group.  AA group had an increased prevalence of metabolic vascular risk factors vs. EA group. However, the role of these factors on CeAD risk have not been elucidated. |
| Gupta et al. 2017 (80) | UK Biobank n = 112.338 of European ancestry  Partners Biobank cohort n= 99 healthy subjects plasma samples | rs9349379(G) minor allele was associated with reduced risk of cervical dissection, migraine and fibromuscular dysplasia, but increased risk of coronary artery disease.  Data from homozygous clones of stem cell lines revealed that rs9349379(G) minor allele increases *EDN1* expression.  The analysis of plasma samples showed an association between rs9349379(G) and higher Big ET-1 levels. |
| Grond-Ginsbach et al. 2017 (62) | CeAD= 833 Europeans without Ehlers-Danlos syndrome  Control groups n= 2.040  Non-CeAD ischemic stroke CTL n = 565  Healthy Finns CADISP-CTL n=215  Healthy Germans PopGen bank CTL n = 1260 | 137 CeAD carriers with 147 rare CNVs affecting 433 protein coding genes. These CNVs were associated with muscle organ development or cell differentiation.  Rare CNVs associated with cardiovascular system development and cell differentiation were carried by 22 CeAD patients, particularly those with family history of CeAD |
| Grond-Ginsbach et al. 2017 (63) | CeAD patients with family history (9 families including 2 members)  Carotid dissection n= 6 families (both members)  Multiple o recurrent dissection n=5 families | 1242 SNVs were found, of those 142 SNVs lead to nonsense, stop-loss or missense substitution.  9 non-benign missense mutations were associated with arterial connective tissue disorders (mutations in *COL3A1*, *FBN1*, *COL4A1*, *TGFBR2* each found in 4 of the 9 families).  Non-benign SNPs associated with connective tissue disorder had increased risk for familial CeAD. |
| Henkin et al. 2016 (6) | SCAD patients n = 59 (out of 116)  M=3 F=54  Age: 44.5±7.9 years. | Disease-causing mutations in 3 patients: FBN1 mutation in a previously diagnosed Marfan syndrome patient: *COL3A1* mutations in 2 patients consistent with Ehlers-Danlos syndrome. Among 12 patients, there were variants of unknown significance but none  was disease-causing. |
| Debette et al.2015 (55) | CeAD groups n=1.393    CeAD-1= 942 (Finns=170 /non-Finnish Europeans= 772)  M=544 F=398  Age: 44.2± 44.6 years    CeAD-2= 451 (Europeans)  M=264 F=188  Age: 44.6 ± 10.5 years  Control groups n= 14.999    Non-CeAD ischemic stroke CLT  n= 583  (Finns=162 /non-Finnish Europeans= 421)  M=257 F=326  Age: 44.6 ± 10.5 years    Healthy CLT n= 14.416 (Finns=287 /non-Finnish Europeans= 14.129)    Follow up study:  CeAD: 659  M=387 F=272  Age: 43.6 ± 9.4 years  CTL: 998 | rs1466535 (*LRP1*), rs6820391(*LNX1*) and rs9349379(G) (*PHACTR1*) SNPs were associated with CeAD. The follow up study showed significant association for rs9349379 in *PHACTR1* with CeAD.   SNPs related to Migraine without aura ( rs9349379 in *PHACTR1*, rs11172113 in LRP1 and rs13208321 in *FHL5*) were associated with CeAD. |
| Grond-Ginsbach et al. 2012 (61) | CeAD groups n=70  M= 46 F = 24  Age: 42.5 ± 9.8 years    CeAD dermal connective tissue alterations (CeAD EM+) n=49  CeAD normal connective tissue (CeAD EM-) n=21  CLT= 403  European origin | 34 CNVs were associated with CeAD from which 18 contained coding sequences mainly in the CeAD EM+ group.  No significant difference in gene density EM vs. CLT.  CNVs in CeAD groups were related to extracellular matrix-collagen fibril organization and TGF-beta signaling. |
| Jara-Prado  et al. 2010 (53) | Case and controls n =144  CeAD n= 48  M= 27 F= 21  Age: 38 ± 10.7 years    CTL: n= 96  M= 54 F=42  Age: 37.8± 10.17 years    Meta-analysis n = 564  CeAD: n=231  CTL n=333    Mexican origin | No significant difference in allelic frequencies for *MTHFR-C677T*, FII G20210A, FV Leiden G1691A, *NOS3* intron 4 a/b VNTR, APOE ε4 gene polymorphisms in CeAD vs. CTL.    No significant association between *MTHFR-C677T* and CeAD. |
| Buss et al. 2009 (54) | CeAD n=70  (Carotid artery n=54; Vertebral artery n=16)  M=42, F= 28  Age: 48.5 years (28-70)    CTL: n= 87  M= 50, F= 37  Age: 48 years (22-68) | No association between matrix metalloproteinases polymorphism and CeAD. No difference in allelic frequencies in CeAD vs. CTL. |

CeAD: cervical artery dissections; CNVs: copy number variants; CTL: control group; SCAD:  spontaneous coronary artery dissections; SNPs: single Nucleotide polymorphism; ADAMTSL4 : ADAMTS Like 4 gene; APOE ε4: Apolipoprotein E  ε4 allele; COL3A1: Collagen Type III Alpha 1 Chain gene; COL4A1: Collagen Type IV Alpha 1 Chain gene; EDN1: endothelin-1 gene; ET-1: endothelin-1 protein; FII G20210A: prothrombin G2021A factor II mutation; *FHL5*: Four And A Half LIM Domains 5 gene; LMX1B: LIM Homeobox Transcription Factor 1 Beta gene; LNX1: Ligand Of Numb-Protein X 1 gene; LRP1: LDL Receptor Related Protein 1 gene; NOS3: Nitric Oxide Synthase 3 gene; PHACTR1: Phosphatase And Actin Regulator 1 gene; SMAD3: SMAD family member 3 gene; TGFBR1: Transforming Growth Factor Beta Receptor 1 gene; TGFBR2: Transforming Growth Factor Beta Receptor 2 gene; WES: whole exome sequencing.
